# Sex differences in the association of sphingolipids with age in Dutch and South-Asian Surinamese living in Amsterdam, the Netherlands

**DOI:** 10.1186/s13293-020-00353-0

**Published:** 2021-01-13

**Authors:** Mirthe Muilwijk, Nardie Callender, Susan Goorden, Frédéric M. Vaz, Irene G. M. van Valkengoed

**Affiliations:** 1 Amsterdam UMC, University of Amsterdam, Department of Public and Occupational Health, Amsterdam Public Health Research Institute, Meibergdreef 9, Amsterdam, The Netherlands; 2 Amsterdam UMC, University of Amsterdam, Laboratory Genetic Metabolic Diseases, Meibergdreef 9, Amsterdam, The Netherlands

**Keywords:** Sphingolipids, Ceramides, Metabolomics, Sex differences, Epidemiology, Dutch, South Asian, HELIUS study

## Abstract

**Background:**

Men have a higher risk for cardiovascular disease (CVD) early in life, while women have a higher risk later in life. The sex-related differences in CVD risk, especially by age, could be related to sphingolipid metabolism. We compared plasma sphingolipid concentrations and its increase by age in men and women.

**Methods:**

Plasma concentrations of 13 types of sphingolipids were measured by liquid chromatography-tandem mass spectrometry in a random subsample of 328 men and 372 women of Dutch and South-Asian Surinamese ethnic origin, participating in the HELIUS study. Sphingolipid concentrations were compared between men and women by age group (18–39, 40–55, and 56–70 years). Multiple linear regression was used to determine sex differences in age trends in sphingolipids stratified by ethnicity. Analyses were performed without adjustment and adjusted for body mass index (BMI) and waist circumference.

**Results:**

At age 18–39 years, sphingolipid concentrations were lower in women than those in men, but at age 56–70 years this was reversed. At higher age, women showed higher concentrations than men. In line, we observed a more rapid increase of sphingolipid concentrations by age in women than in men. The observed sex differences were not explained by BMI or waist circumference. Patterns of sex differences were similar across ethnic groups, although the strength of associations differed.

**Conclusions:**

Mean sphingolipid concentrations increase more rapidly with age in women than in men. Therefore, plasma lipid concentrations of sphingolipids, although lower in women than in men at younger age, are higher in women than in men at older age.

**Supplementary Information:**

The online version contains supplementary material available at 10.1186/s13293-020-00353-0.

## Introduction

Each year, 41 million people die from non-communicable diseases (NCDs) globally, this accounts for 71% of all deaths [1]. Two major classes of NCDs are cardiovascular diseases (CVDs) and type 2 diabetes (T2D) [1], which both show different distributions in prevalence between men and women [2]. Men, for instance, have a higher risk for CVD early in life, while women have a higher risk later in life [3, 4]. Despite these observed differences, mechanisms potentially linking these sex-related differences to CVD risk remain understudied.

Mechanisms related to the previously observed sex differences in body fat may partly explain observed differences in prevalence of CVD and T2D. Men tend to store higher amounts of ectopic and visceral fat than women, while women are relatively protected from ectopic fat storage [5, 6]. With age, hormonal changes occur, such as a drop in oestrogen levels in women after menopause. These hormonal changes affect deposition of ectopic and visceral fat [7]. While both men and women tend to store higher amounts of visceral fat with increasing age, the amount of visceral fat is shown to increase by 200% in men, but with 400% in women [8].

A mechanism that may play a role is the increased formation of potentially toxic lipid intermediates such as sphingolipids, due to the increase of ectopic fat. The higher availability of free fatty acids increases for example ceramide (a class of sphingolipids) synthesis. Sphingolipid levels have been shown to be associated with various diseases [9–12], including CVD [13–17], T2D [18–23], and metabolic syndrome [24, 25]. Where long-chain (dihydro) ceramides have been positively associated with T2D and CVD risk, very-long-chain ceramides and more complex sphingolipids including lactosylceramides have been negatively associated [23, 26]. Moreover, enzymes of sphingolipid synthesis are potential targets to reduce CVD risk [27], and the inhibition of glycosphingolipid biosynthesis has for instance been shown to decrease atherosclerosis in mice [28]. Previous studies already showed that men have higher levels of circulating ceramides than premenopausal women [29], while ceramide levels increase more rapidly in post-menopausal women than in men [30]. Sphingolipids could, thus, potentially explain not only the higher prevalence of CVD in men compared to women, but also the accelerated occurrence of CVD in post-menopausal women. How other classes of sphingolipids, e.g., more complex glucosylceramides and lactosylceramides, increase by age in both men and women has not been determined yet.

In our cross-sectional study, we describe the differences in mean sphingolipid concentrations across age groups between 18- and 70-year-old Dutch and South-Asian Surinamese men and women living in Amsterdam, the Netherlands. In addition, we explored age trends in sphingolipids and determined whether age trends in sphinogolipids differ by sex.

## Materials and methods

### Population

Baseline data from the Healthy Life in an Urban Setting (HELIUS) study, collected between 2011 and 2015, was used. HELIUS is a multi-ethnic cohort study among six ethnic groups living in Amsterdam. A detailed description of the design is available elsewhere [31, 32]. In brief, participants were randomly sampled from the municipal register, stratified by ethnicity. Questionnaires, physical examinations, and biological samples were obtained [31]. Full data were collected among 22,165 participants, from whom we selected those of Dutch and South-Asian Surinamese ethnicity (*n* = 7607), because the current study includes secondary analyses of data collected as part of a HELIUS sub-study aimed at studying causes of incident T2D among high-risk South-Asian populations and sphingolipids were not determined in other ethnicities. We then excluded participants who did not provide permission for data linkage or storage of biological material (*n* = 671) and those who had less than two vials of EDTA-plasma available in the biobank (*n* = 186). In addition, participants with T2D based on self-report, increased fasting glucose (≥ 7.0 mmol/L), increased HbA1c (≥ 48 mmol/mol), or use of glucose lowering medication were excluded (*n* = 773). From the 5977 participants (3972 of Dutch and 2005 of South-Asian Surinamese origin) who remained in the study, we took a random sample of 350 participants per ethnic group in whom metabolites were determined using the sample function in the R statistical software package. The Institutional Review Board of the Amsterdam Medical Center approved the HELIUS study (MREC 10/100# 17.10.1729). All participants provided written informed consent.

### Measurements

Ethnicity was defined by the individual’s country of birth combined with the parental countries of birth. Dutch ethnicity was assigned to participants born in the Netherlands, with both parents born in the Netherlands. South-Asian Surinamese ethnicity was assigned to participants born in Suriname with at least one parent born in Suriname (1st generation) or born in the Netherlands with both parents born in Suriname (2nd generation) combined with self-reported South-Asian ethnic origin.

Body mass index (BMI) was determined by dividing measured body weight (kg) by height squared (m^2^). Weight and height were measured in barefoot subjects wearing light clothes only. Waist circumference was measured using a tape measure at the level midway between the lowest rib margin and the iliac crest. All anthropometric measures were taken in duplicate, and the mean was used in the analyses. If the discrepancy between the duplicate measures differed more than 0.5 cm for height, more than 0.5 kg for weight, or more than 1 cm for waist circumference, a third measurement was taken. The two measures which were most similar were used to calculate the mean.

The total reported fat intake and total energy intake were derived from an ethnic-specific food frequency questionnaire (FFQ) which was taken among a subsample of the HELIUS cohort, as described in detail elsewhere [33]. The FFQ data were available for 259 participants of our study sample, of whom 58 participants were Dutch men, 47 South-Asian men, 67 Dutch women, and 87 South-Asian women. Menopause was derived from the questionnaire based on lack of menstruation for a year or longer (not for reasons such as pregnancy, breastfeeding, or using birth control).

Blood was collected after a fasting period of at least 10 h. Sphingolipids were measured in plasma by liquid chromatography-tandem mass spectrometry (LC-tMS) as described previously [23]. We adjusted for amino acids in sensitivity analyses. These were determined in plasma by LC-tMS as described previously [34].

### Statistical analyses

First, the normal distribution of variables was checked by plotting histograms and evaluating skewness and kurtosis. Baseline characteristics and sphingolipid concentrations were examined among men and women stratified by ethnicity. We calculated means and standard deviations (SD) for continuous normally distributed variables, medians, and interquartile ranges for continuous non-normally distributed variables and numbers of observations and percentages for categorical variables. Baseline characteristics were not tested for statistical differences [35]. Waist circumference was missing for one participant and imputed with an expectation-maximization algorithm. Sex differences in sphingolipid concentrations stratified by age (categories 18–39, 40–55, and 56–70 years) were studied by multiple linear regression within each age group.

Second, we analyzed the association of metabolites with age. We checked the linearity of the association by plotting scatterplots in the total population and stratified by ethnicity. The multiplicative interaction of age with ethnicity was checked by adding an interaction term between age and ethnicity with sphingolipids as the outcome. This was done because BMI may reflect different levels of intra-abdominal fat storage in European than South-Asian populations [36], which may also have implications for the use of non-oxidative pathways. A multiplicative interaction between age and ethnicity was observed for five of the thirteen included sphingolipids (GlcCer(d18:2), GlcCer(d18:1), LacCer(d18:2), LacCer(d18:1), and Cer(d18:1)). Analyses were thus stratified by ethnicity in all analyses. A multiplicative interaction term between age and sex was used to investigate whether the association between sphingolipids and age differed by sex.

All models were run both unadjusted and adjusted for measures of body fat distribution (BMI and waist circumference). We adjusted for cholesterol- and blood pressure-lowering medication in sensitivity analyses, as the use may affect sphingolipid concentrations [29, 37]. We also checked whether the amount of substrate available influenced the results, by adjusting for important substrates for sphingolipids including amino acids (serine, alanine, and glycine), and fat and energy intake in the subset of the population with FFQ data available. Finally, we excluded participants with CVD at baseline. In post-hoc analyses, we adjusted for menopause.

All analyses were conducted using IBM SPSS Statistics 23. Graphs were plotted in RStudio version 3.6.1 using the visreg package. Tests were two-sided, and *p* values < 0.05 were considered statistically significant. Analyses were not adjusted for multiple testing as our study was of exploratory nature [38], but the consistency of findings was considered to avoid chance findings.

## Results

Mean age in the 18–39 year group ranged from 28.4 (SD 5.7) among South-Asian Surinamese men to 31.3 (SD 4.8) among Dutch men, that in the 40–55 year group from 46.8 (SD 4.4) among Dutch men to 47.8 (SD 4.3) among South-Asian Surinamese women, and that in the 56–70 year group from 59.9 (SD 4.1) among South-Asian Surinamese women to 62.0 (SD 4.5) among Dutch men. Mean BMI ranged from 22.5 (SD 3.1) in Dutch women aged 18–39 years to 26.7 (SD 4.6) in South-Asian Surinamese women aged 55–70 years (Table 1). Mean waist circumference was lowest among Dutch women aged 18–39 years old with a mean of 79.5 (SD 9.0) and highest in 55–70-year-old men with a mean of 97.6 (SD 11.3).

**Table 1:** baseline characteristics of participants, stratified by sex, ethnicity and age group

	**Dutch men** **18–39 years** **(*n*** **= 59)**	**Dutch men** **40–55 years** **(*n*** **= 57)**	**Dutch men** **56–70 years** **(*n*** **= 58)**	**Dutch women** **18–39 years** **(*n*** **= 68)**	**Dutch women** **40–55 years** **(*n*** **= 55)**	**Dutch women** **56–70 years** **(*n*** **= 53)**
Age (years)	31.3 (4.8)	46.8 (4.4)	62.0 (4.5)	29.4 (5.6)	47.7 (4.4)	61.0 (3.9)
BMI (kg/m^2^)	23.6 (3.9)	25.2 (3.9)	26.1 (3.8)	22.5 (3.1)	24.3 (4.0)	24.8 (3.3)
Waist circumference (cm)	86.9 (12.3)	94.3 (11.2)	97.6 (11.3)	79.5 (9.0)	86.9 (12.2)	88.0 (10.2)
Energy intake (Kcal)^a^	2411 (1937–2900)	2516 (2226–2855)	2377 (1976–2921)	1948 (1425–2424)	1967 (1605–2161)	1792 (1496–2199)
Fatty acids intake (g)^a^	93.1 (79.5–109.1)	92.8 (74.4–92.8)	86.5 (65.7–114.2)	75.3 (49.4–93.0)	74.5 (47.3–85.3)	64.3 (52.8–94.3)
Alanine (μmol/L)	310 (70)	324 (65)	319 (53)	280 (65)	306 (63)	305 (67)
Glycine (μmol/L)	154 (127–154)	144 (124–169)	151 (131–184)	145 (112–180)	160 (131–202)	182 (149–213)
Serine (μmol/L)	91 (83–103)	89 (80–99)	92 (83–102)	96 (85–112)	91 (82 - 107)	99 (84–108)
Menopause (%)	-	-	-	0.0 (0)	25.5 (14)	96.2 (51)
Cholesterol lowering medication (%)	0.0 (0)	3.5 (2)	12.1 (7)	0.0 (11)	0.0 (0)	5.6 (13.2)
Blood pressure medication (%)	0.0 (0)	3.5 (2)	27.6 (16)	2.9 (2)	3.6 (2)	18.9 (10)
	**SA Sur men** **18**–**39 years** **(*n*** **= 53)**	**SA Sur men** **40**–**55 years** **(*n*** **= 75)**	**SA Sur men** **56**–**70 years** **(*n*** **= 26)**	**SA Sur women** **18**–**39 years** **(*n*** **= 63)**	**SA Sur women** **40**–**55 years** **(*n*** **= 84)**	**SA Sur women** **56**–**70 years** **(*n*** **= 49)**
Age (years)	28.4 (5.7)	47.0 (4.0)	61.5 (4.3)	28.5 (5.7)	47.8 (4.3)	59.9 (4.1)
BMI (kg/m^2^)	25.0 (3.9)	25.6 (3.3)	25.9 (3.7)	24.9 (5.4)	26.1 (4.5)	26.7 (4.6)
Waist circumference (cm)	89.7 (11.5)	93.1 (10.7)	96.0 (9.2)	83.8 (14.5)	88.5 (11.0)	92.6 (12.6)
Energy intake (Kcal)^a^	2585 (1381–3092)	2099 (1772–2491)	2168 (1917–3224)	1933 (1228–2212)	1794 (1531–2168)	1761 (1296–1959)
Fatty acids intake (g)^a^	81.6 (39.2–117.3)	62.1 (56.2–82.5)	64.2 (56.5–100.4)	58.2 (39.6–74.4)	57.2 (47.3–76.5)	50.1 (38.7–69.2)
Alanine (μmol/L)	339 (79)	353 (78)	355 (63)	316 (80)	319 (65)	341 (60)
Glycine (μmol/L)	131 (119–154)	138 (120–159)	139 (127–163)	138 (115–176)	145 (118–177)	155 (126–193)
Serine (μmol/L)	95 (87–108)	96 (87–108)	85 (76–99)	100 (84–111)	95 (80–109)	94 (77–107)
Menopause (%)	-	-	-	4.8 (3)	26.2 (22)	81.6 (40)
Cholesterol lowering medication (%)	3.8 (2)	20.0 (15)	46.2 (12)	0.0 (0)	2.4 (2)	18.4 (9)
Blood pressure medication (%)	1.9 (1)	17.3 (13)	53.8 (14)	3.2 (2)	13.1 (11)	38.8 (19)

Data are mean (SD), median (IQR), or % (*n*). *SA Sur* South-Asian Surinamese

^a^Available for a subset of the population (*n* = 13 Dutch men 18–39 years, *n* = 17 Dutch men 40–55 years, *n* = 28 Dutch men 56–70 years, *n* = 24 Dutch women 18–39 years, *n* = 22 Dutch women 40–55 years, *n* = 21 Dutch women 56–70 years, *n* = 11 South-Asian Surinamese men 18–39 years, *n* = 27 South-Asian Surinamese men 40–55 years, *n* = 9 South-Asian Surinamese men 56–70 years, *n* = 24 South-Asian Surinamese women 18–39 years, *n* = 39 South-Asian Surinamese women 40–55 years, *n* = 24 South-Asian Surinamese women 56–70 years)

Dihydroceramide (Cer(d20:1), Cer(d18:2), Cer(d18:1), Cer(d18:0), Cer(d17:1), and Cer(d16:1)) concentrations were generally lower in women than in men in the 18–39 years age group, although mostly not statistically significantly different (Table 2). The sphingolipid concentrations were generally, however, statistically significantly higher in women than in men in the older age groups, especially in the 56–70 years group. The age-adjusted difference in women compared to that in men for Cer(d18:2) was for instance − 123.4 (95% CI − 244; − 2.3) nmol/L in the 18–39 years age group and 208.2 (95% CI 37.2; 379.2) nmol/L in the 56−70 years age group. The more complex sphingolipids (GlcCer(d18:2), GlcCer(d18:2), LacCer(d18:2), LacCer(d18:1), CTH(d18:1), and CTH(d20:1)) showed similar patterns, but were already higher in women in the 18− 39 years age groups in the South-Asian Surinamese with a further increase in the difference with men in older age groups. Patterns of sex differences in mean sphingolipid concentrations remained similar after adjustment for BMI and waist circumference.

**Table 2:** Baseline sphingolipid concentrations in men compared to women, stratified by age group

**Cer(d18:1) (nmol/L)**							
		**Men**	**Women**	**Age-adjusted difference**		**Age-, BMI-, and waist-adjusted difference**	
		**Mean (SD)**	**Mean (SD)**	**B (95% CI)**	***P*** **value**	**B (95% CI)**	***P*** **value**
**Dutch**	18–39 years (*N* = 59/68)	8369 (2095)	7572 (1371)	– 707 (– 1327; – 86)	***0.03***	– 432 (– 1081; 217)	0.19
	40–55 years (*N* = 57/55)	9562 (2413)	8957 (2417)	– 744 (– 1621; 133)	0.10	– 386 (– 1346; 574)	0.43
	56–70 years (*N* = 58/53)	9926 (2435)	10244 (1874)	324 (– 509; 1157)	0.44	512 (– 464; 1489)	0.30
**South-Asian Surinamese**	18–39 years (*N* = 53/63)	8311 (2186)	7689 (1712)	– 625 (– 1330; 80)	0.08	– 259 (– 1076; 558)	0.53
	40–55 years (*N* = 75/84)	8964 (2006)	8631 (2106)	– 407 (– 1047; 232)	0.21	– 539 (– 1274; 196)	0.15
	56–70 years (*N* = 26/49)	8476 (2364)	8581 (1695)	170 (– 790; 1130)	0.73	105 (– 982; 1192)	0.85
**Cer(d18:2) (nmol/L)**							
		**Men**	**Women**	**Age-adjusted difference**		**Age-, BMI-, and waist-adjusted difference**	
		**Mean (SD)**	**Mean (SD)**	**B (95% CI)**	***P*** **value**	**B (95% CI)**	***P*** **value**
**Dutch**	18–39 years (*N* = 59/68)	1256 (423)	1113 (250)	– 123 (– 245; – 2)	***0.05***	– 87 (– 215; 41)	0.18
	40–55 years (*N* = 57/55)	1397 (427)	1364 (448)	– 55 (– 216; 106)	0.50	2 (– 175; 179)	0.98
	56–70 years (*N* = 58/53)	1448 (438)	1653 (461)	208 (37; 379)	***0.02***	204 (3; 406)	***0.05***
**South-Asian Surinamese**	18–39 years (*N* = 53/63)	1222 (405)	1221 (350)	– 1 (– 138; 136)	0.99	94 (– 64; 252)	0.24
	40–55 years (*N* = 75/84)	1351 (424)	1386 (449)	23 (– 114; 160)	0.74	– 37 (– 194; 119)	0.64
	56–70 years (*N* = 26/49)	1374 (448)	1551 (358)	181 (– 13; 374)	0.07	153 (– 66; 372)	0.17
**Cer(d18:0) (nmol/L)**							
		**Men**	**Women**	**Age-adjusted difference**		**Age-, BMI-, and waist-adjusted difference**	
		**Mean (SD)**	**Mean (SD)**	**B (95% CI)**	***P*** **value**	**B (95% CI)**	***P*** **value**
**Dutch**	18–39 years (*N* = 59/68)	2081 (610)	2185 (673)	126 (– 105; 357)	0.28	260 (22; 498)	***0.03***
	40–55 years (*N* = 57/55)	2240 (551)	2287 (568)	18 (– 187; 222)	0.87	84 (– 138; 305)	0.46
	56–70 years (*N* = 58/53)	2305 (552)	2491 (504)	– 13 (– 37; 373)	0.09	29 (59; 525)	***0.01***
**South-Asian Surinamese**	18–39 years (*N* = 53/63)	2264 (583)	2229 (573)	– 36 (– 249; 178)	0.74	– 96 (– 340; 149)	0.44
	40–55 years (*N* = 75/84)	2415 (723)	2480 (679)	54 (– 167; 274)	0.63	58 (– 194; 310)	0.65
	56–70 years (*N* = 26/49)	2316 (754)	2285 (601)	– 14 (– 338; 311)	0.93	– 119 (– 491; 252)	0.52
**Cer(d16:1) (nmol/L)**							
		**Men**	**Women**	**Age-adjusted difference**		**Age-, BMI-, and waist-adjusted difference**	
		**Mean (SD)**	**Mean (SD)**	**B (95% CI)**	***P*** **value**	**B (95% CI)**	***P*** **value**
**Dutch**	18–39 years (*N* = 59/68)	449 (192)	418 (143)	– 25 (– 85; 35)	0.40	3 (– 60; 65)	0.93
	40–55 years (*N* = 57/55)	537 (222)	476 (162)	– 72 (– 143; – 2)	***0.05***	– 63 (– 141; 15)	0.11
	56–70 years (*N* = 58/53)	541 (206)	601 (219)	64 (– 17; 145)	0.12	72 (– 23; 166)	0.14
**South-Asian Surinamese**	18–39 years (*N* = 53/63)	422 (208)	407 (156)	– 15 (– 81; 50)	0.64	23 (– 53; 99)	0.55
	40–55 years (*N* = 75/84)	466 (167)	457 (163)	– 13 (– 65; 39)	0.62	– 34 (– 93; 26)	0.27
	56–70 years (*N* = 26/49)	536 (257)	529 (166)	9.0 (– 86; 104)	0.85	– 8 (– 115; 100)	0.89
**Cer(d17:1) (nmol/L)**							
		**Men**	**Women**	**Age-adjusted difference**		**Age-, BMI-, and waist-adjusted difference**	
		**Mean (SD)**	**Mean (SD)**	**B (95% CI)**	***P*** **value**	**B (95% CI)**	***P*** **value**
**Dutch**	18–38 years (*N* = 59/68)	369 (109)	343 (102)	– 19 (– 57; 18)	0.30	– 16 (– 57; 25)	0.44
	40–55 years (*N* = 57/55)	430 (135)	394 (122)	– 42 (– 89; 6)	0.08	– 39 (– 91; 14)	0.15
	56–70 years (*N* = 58/53)	438 (165)	475 (132)	37 (– 21; 94)	0.21	33 (– 35; 101)	0.34
**South-Asian Surinamese**	18–39 years (*N* = 53/63)	307 (115)	296 (89)	– 11 (– 48; 26)	0.56	6 (– 38; 49)	0.80
	40–55 years (*N* = 75/84)	333 (112)	324 (114)	– 14 (– 49; 22)	0.45	– 33 (– 73; 7)	0.11
	56–70 years (*N* = 26/49)	347 (145)	356 (93)	17 (– 38; 72)	0.53	2 (– 58; 61)	0.96
**Cer(d20:1) (nmol/L)**							
		**Men**	**Women**	**Age-adjusted difference**		**Age-, BMI-, and waist-adjusted difference**	
		**Mean (SD)**	**Mean (SD)**	**B (95% CI)**	***P*** **value**	**B (95% CI)**	***P*** **value**
**Dutch**	18–39 years (*N* = 59/68)	186 (62)	192 (155)	13 (– 30; 56)	0.55	17 (– 30; 64)	0.47
	40–55 years (*N* = 57/55)	211 (67)	189 (65)	– 26 (– 50; – 2)	***0.03***	– 23 (– 50; 4)	0.09
	56–70 years (*N* = 58/53)	218 (69)	207 (55)	– 10 (– 34; 14)	0.40	– 1 (– 29; 27)	0.93
**South-Asian Surinamese**	18–39 years (*N* = 53/63)	189 (73)	155 (48)	– 34 (– 56; – 12)	***0.003***	– 28 (-54; – 1)	***0.04***
	40–55 years (*N* = 75/84)	201 (80)	179 (82)	– 23 (49; 21)	0.07	– 31 (– 59; – 2)	***0.04***
	56–70 years (*N* = 26/49)	165 (50)	178 (71)	10 (– 22; 41)	0.55	11 (– 25; 47)	0.55
**Cer(m18:0) (nmol/L)**							
		**Men**	**Women**	**Age-adjusted difference**		**Age-, BMI-, and waist-adjusted difference**	
		**Mean (SD)**	**Mean (SD)**	**B (95% CI)**	***P*** **value**	**B (95% CI)**	***P*** **value**
**Dutch**	18–39 years (*N* = 59/68)	31 (12)	26 (8)	– 5 (– 9; – 2)	***0.005***	– 3 (– 6; 1)	0.14
	40–55 years (*N* = 57/55)	33 (12)	29 (11)	– 4 (– 9;0)	0.05	– 2 (– 7; 3)	0.41
	56–70 years (*N* = 58/53)	33 (11)	31 (11)	– 2 (– 6; 2)	0.27	2 (– 3; 6)	0.52
**South-Asian Surinamese**	18–39 years (*N* = 53/63)	33 (14)	26 (11)	– 7 (– 12; – 3)	***0.003***	– 5 (– 11; 0)	***0.05***
	40–55 years (*N* = 75/84)	35 (16)	30 (12)	– 5 (– 10; – 1)	***0.02***	– 4 (– 9; 1)	0.08
	56–70 years (*N* = 26/49)	36 (12)	31 (14)	– 5 (– 11; 2)	0.17	– 5 (– 12; 3)	0.21
**GlcCer(d18:1) (nmol/L)**							
		**Men**	**Women**	**Age-adjusted difference**		**Age-, BMI-, and waist-adjusted difference**	
		**Mean (SD)**	**Mean (SD)**	**B (95% CI)**	***P*** **value**	**B (95% CI)**	***P*** **value**
**Dutch**	18–39 years (*N* = 59/68)	4265 (1073)	3899 (990)	– 300 (– 665; 64)	0.11	– 228 (– 624; 169)	0.26
	40–55 years (*N* = 57/55)	4481 (1045)	4353 (964)	– 153 (– 531; 224)	0.42	– 254 (– 682; 173)	0.24
	56–70 years (*N* = 58/53)	4870 (1108)	4963 (1463)	132 (– 355; 619)	0.59	17 (– 534; 569)	0.95
**South-Asian Surinamese**	18–39 years (*N* = 53/63)	3878 (932)	3963 (859)	84 (– 244; 413)	0.61	138 (– 251; 526)	0.48
	40–55 years (*N* = 75/84)	3970 (1041)	4198 (1044)	229 (– 100; 559)	0.17	171 (– 209; 550)	0.38
	56–70 years (*N* = 26/49)	3873 (1366)	3953 (878)	157 (– 361; 676)	0.55	215 (– 362; 791)	0.46
**GlcCer(d18:2) (nmol/L)**							
		**Men**	**Women**	**Age-adjusted difference**		**Age-, BMI-, and waist-adjusted difference**	
		**Mean (SD)**	**Mean (SD)**	**B (95% CI)**	***P*** **value**	**B (95% CI)**	***P*** **value**
**Dutch**	18–39 years (*N* = 59/68)	528 (147)	507 (124)	– 13 (– 61; 35)	0.59	– 8 (– 60; 45)	0.78
	40–55 years (*N* = 57/55)	558 (138)	611 (159)	27 (– 28; 82)	0.33	15 (– 47; 77)	0.47
	56–70 years (*N* = 58/53)	652 (160)	734 (216)	86 (15; 158)	***0.02***	70 (– 13; 152)	0.10
**South-Asian Surinamese**	18–39 years (*N* = 53/63)	483 (126)	550 (122)	68 (22; 113)	***0.004***	73 (19; 127)	***0.009***
	40–55 years (*N* = 75/84)	506 (142)	614 (185)	106 (54; 159)	***< 0.001***	86 (26; 146)	***0.005***
	56–70 years (*N* = 26/49)	526 (175)	648 (137)	136 (64; 209)	***< 0.001***	144 (64; 224)	***0.001***
**LacCer(d18:1) (nmol/L)**							
		**Men**	**Women**	**Age-adjusted difference**		**Age-, BMI-, and waist-adjusted difference**	
		**Mean (SD)**	**Mean (SD)**	**B (95% CI)**	***P*** **value**	**B (95% CI)**	***P*** **value**
**Dutch**	18–39 years (*N* = 59/68)	3458 (857)	3311 (732)	– 132 (– 418; 152)	0.36	– 120 (– 431; 191)	0.45
	40–55 years (*N* = 57/55)	3372 (765)	3533 (740)	169 (– 116; 454)	0.24	114 (– 207; 435)	0.48
	56–70 years (*N* = 58/53)	3457 (752)	3583 (948)	149 (– 174; 471)	0.36	0 (– 360; 360)	1.00
**South-Asian Surinamese**	18–39 years (*N* = 53/63)	3115 (765)	3308 (716)	193 (– 81; 467)	0.17	273 (– 50; 595)	0.10
	40–55 years (*N* = 75/84)	2970 (727)	3238 (706)	286 (61; 511)	***0.01***	287 (28; 546)	***0.03***
	56–70 years (*N* = 26/49)	2874 (935)	2889 (642)	19 (– 355; 392)	0.92	– 13 (– 433; 407)	0.95
**LacCer(d18:2) (nmol/L)**							
		**Men**	**Women**	**Age-adjusted difference**		**Age-, BMI-, and waist-adjusted difference**	
		**Mean (SD)**	**Mean (SD)**	**B (95% CI)**	***P*** **value**	**B (95% CI)**	***P*** **value**
**Dutch**	18–39 years (*N* = 59/68)	452 (117)	438 (98)	– 10 (– 48; 28)	0.61	– 4 (– 46; 38)	0.86
	40–55 years (*N* = 57/55)	461 (106)	526 (125)	61 (18; 105)	***0.006***	55 (7; 103)	***0.03***
	56–70 years (*N* = 58/53)	492 (112)	584 (161)	95 (43; 147)	***< 0.001***	80 (21; 140)	***0.009***
**South-Asian Surinamese**	18–39 years (*N* = 53/63)	395 (97)	458 (106)	66 (25; 100)	***0.001***	78 (34; 123)	***0.001***
	40–55 years (*N* = 75/84)	386 (97)	491 (110)	104 (71; 137)	***< 0.001***	100 (62; 138)	***< 0.001***
	56–70 years (*N* = 26/49)	396 (108)	495 (110)	102 (48; 156)	***< 0.001***	101 (41; 161)	***0.001***
**CTH(d18:1) (nmol/L)**							
		**Men**	**Women**	**Age-adjusted difference**		**Age-, BMI-, and waist-adjusted difference**	
		**Mean (SD)**	**Mean (SD)**	**B (95% CI)**	***P*** **value**	**B (95% CI)**	***P*** **value**
**Dutch**	18–39 years (*N* = 59/68)	1004 (270)	1053 (306)	62 (– 41; 166)	0.23	66 (– 46; 178)	0.25
	40–55 years (*N* = 57/55)	1023 (239)	1100 (263)	78 (– 17; 173)	0.11	38 (– 69; 145)	0.48
	56–70 years (*N* = 58/53)	1067 (259)	1218 (385)	160 (37; 283)	***0.01***	89 (– 47; 226)	0.20
**South-Asian Surinamese**	18–39 years (*N* = 53/63)	950 (258)	1084 (244)	133 (40; 226)	***0.005***	117 (7; 228)	***0.04***
	40–55 years (*N* = 75/84)	940 (247)	1110 (244)	170 (93; 248)	***< 0.001***	138 (51; 225)	***0.002***
	56–70 years (*N* = 26/49)	958 (241)	1109 (265)	164 (38; 290)	***0.01***	164 (32; 297)	***0.02***
**CTH(d18.2) (nmol/L)**							
		**Men**	**Women**	**Age-adjusted difference**		**Age-, BMI-, and waist-adjusted difference**	
		**Mean (SD)**	**Mean (SD)**	**B (95% CI)**	***P*** **value**	**B (95% CI)**	***P*** **value**
**Dutch**	18–39 years (*N* = 59/68)	204 (53)	234 (67)	318 (10; 54)	***0.005***	35 (11; 59)	***0.005***
	40–55 years (*N* = 57/55)	215 (57)	262 (76)	46 (21; 71)	***< 0.001***	39 (11; 67)	***0.008***
	56–70 years (*N* = 58/53)	235 (57)	292 (90)	59 (31; 87)	***< 0.001***	42 (10; 75)	***0.01***
**South-Asian Surinamese**	18–39 years (*N* = 53/63)	208 (53)	274 (71)	66 (46; 89)	***< 0.001***	64 (36; 91)	***< 0.001***
	40–55 years (*N* = 75/84)	206 (61)	285 (69)	79 (58; 99)	***< 0.001***	69 (45; 92)	***< 0.001***
	56–70 years (*N* = 26/49)	223 (53)	318 (87)	99 (61; 137)	***< 0.001***	100 (57; 143)	***< 0.001***

**Fig. 1:**
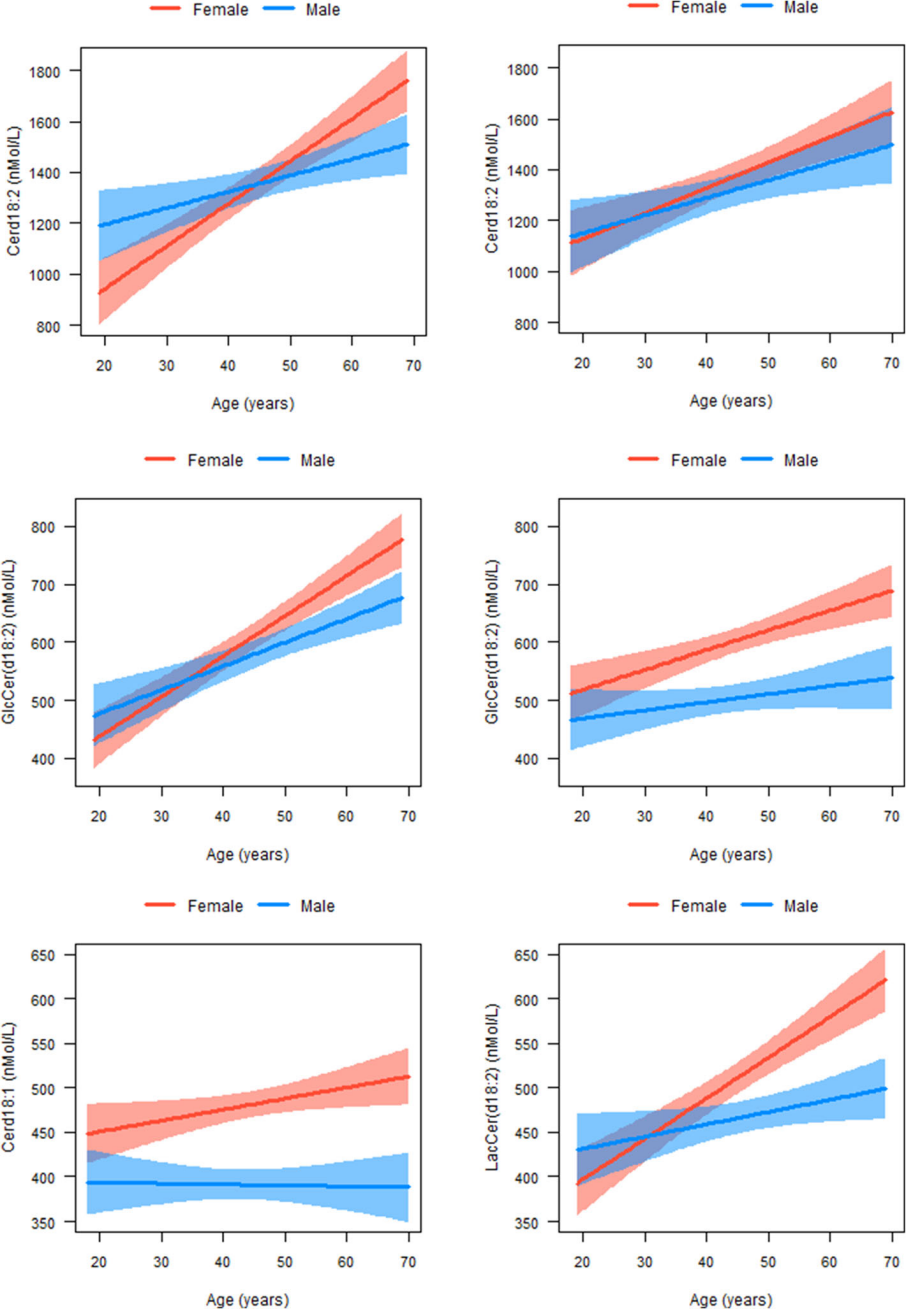
Sphingolipids which concentrations increase faster by age in women than men. The sphingolipids are shown of which the plasma concentrations increase faster by age in women than in men. A significant interaction by sex means a statistically significant multiplicative interaction between age and sex with sphingolipid concentrations as the outcome at a *P* value < 0.05. Analyses were stratified by ethnicity. Red asterisk denotes statistically significant association between age and sphingolipid in women at a *P* value < 0.05. Blue asterisk denotes statistically significant association between age and sphingolipid in men a *P* value < 0.05

Most sphingolipids increased with age in both men and women (Fig. 1; Table 3). Cer(d18:1) for instance increased with 52.28 nmol/L (95% CI 26.56; 78.00) per year in Dutch men. However, no clear trends were observed for CTH(d18:1) and LacCer(d18:1). Most plasma concentrations of sphingolipids increased more with age in women than in men, although only statistically significantly differed for GlcCer(d18:2), LacCer(d18:2), and Cer(d18:2). Figure 1 shows that plasma concentrations of sphingolipids are generally lower in young adult women than in men, but higher in women than in men from the age of approximately 45 years. The patterns in the associations of sphingolipids and age did not change after adjusting for BMI and waist circumference. Although the strength of the associations differed by ethnicity, patterns of differences in age trends between men and women were similar.

**Table 3:** Association of sphingolipids with age

Sphingolipid (nMol/L)	Dutch men (***N*** = 174)		Dutch women (***N*** = 176)		Interaction by sex	SA Sur men (***N*** = 154)		SA Sur women (***N*** = 196)		Interaction by sex
	B (95% CI)	***P*** value	B (95% CI)	***P*** value	***P*** value	B (95% CI)	***P*** value	B (95% CI)	***P*** value	***P*** value
**Ceramides**										
Cer(d18:1)										
Model 1	52 (27; 78)	**< 0.001**	82 (62; 102)	**< 0.001**	0.07	27 (0; 54)	**0.05**	33 (13; 53)	**0.002**	0.72
Model 2	31 (2; 59)	**0.03**	76 (55; 98)	**< 0.001**	**0.05**	33 (4; 61)	**0.03**	31 (10; 53)	**0.005**	0.73
Cer(d18:2)										
Model 1	6.4 (1.6; 11.2)	**0.009**	16.7 (12.7; 20.7)	**< 0.001**	**0.001**	6.9 (1.7; 12.2)	**0.01**	9.9 (5.6; 14.2)	**< 0.001**	0.38
Model 2	3.2 (− 2.1; 8.5)	0.23	15.92 (11.57; 20.26)	**< 0.001**	**0.001**	8.3 (2.7; 14.0)	**0.004**	9.9 (5.4 14.52)	**< 0.001**	0.37
Cer(d18:0)										
Model 1	7.9 (1.5; 14.2)	**0.02**	9.4 (3.1; 15.7)	**0.004**	0.73	5.0 (− 3.6; 13.6)	0.25	4.1 (− 2.8; 11.0)	0.24	0.87
Model 2	2.2 (− 4.7; 9.1)	0.53	5.7 (− 1.0; 12.3)	0.09	0.55	6.6 (− 2.4; 15.7)	0.15	3.2 (− 4.2; 10.5)	0.40	0.70
Cer(d16:1)										
Model 1	3.2 (0.9; 5.5)	**0.007**	5.7 (3.9; 7.5)	**< 0.001**	0.10	4.3 (1.9; 6.8)	**0.001**	3.6 (1.9; 5.3)	**< 0.001**	0.62
Model 2	1.1 (− 1.3; 3.6)	0.37	5.3 (3.4; 7.3)	**< 0.001**	0.07	4.7 (2.1; 7.3)	**< 0.001**	3.8 (1.9; 5.6)	**< 0.001**	0.59
Cer(d16:1)										
Model 1	2.3 (0.8; 3.9)	**0.003**	4.0 (2.7; 5.2)	**< 0.001**	0.10	2.0 (0.5; 3.4)	**0.01**	2.0 (0.9; 3.1)	**< 0.001**	0.97
Model 2	1.7 (− 0.0; 3.4)	0.06	4.1 (2.7; 5.4)	**< 0.001**	0.10	2.4 (0.8; 4.0)	**0.003**	2.3 (1.1; 3.4)	**< 0.001**	0.96
Cer(d20:1)										
Model 1	1.07 (0.33; 1.81)	**0.005**	0.86 (− 0.27; 1.99)	0.13	0.77	− 0.07 (− 1.01; 0.87)	0.88	0.75 (− 0.01; 1.51)	0.05	0.18
Model 2	0.79 (− 0.04; 1.61)	0.06	0.88 (− 0.34; 2.11)	0.16	0.85	0.09 (− 0.91; 1.09)	0.86	0.71 (− 0.10; 1.53)	0.09	0.21
**1-Deoxysphinganine**										
Cer(m18:0)										
Model 1	7.9 (1.5; 14.2)	**0.02**	9.4 (3.1; 15.7)	**0.004**	0.73	5.0 (− 3.6; 13.6)	0.25	4.1 (− 2.8; 11.0)	0.24	0.87
Model 2	2.2 (− 4.7; 9.1)	0.53	5.7 (− 1.0; 12.3)	0.09	0.55	6.6 (− 2.4; 15.7)	0.15	3.2 (− 4.2; 10.5)	0.40	0.70
**Glucosylceramides**										
GlcCer(d18:1)										
Model 1	21 (9; 33)	**0.001**	33 (21; 45)	**< 0.001**	0.16	3 (− 10; 17)	0.64	4 (− 6; 14)	0.43	0.91
Model 2	21 (8; 34)	**0.002**	40 (27; 52)	**< 0.001**	0.17	6 (− 9; 20)	0.44	5 (− 6; 16)	0.38	0.52
GlcCer(d18:2)										
Model 1	4.1 (2.4; 5.7)	**< 0.001**	6.9 (5.2; 8.7)	**< 0.001**	**0.02**	1.4 (− 0.4; 3.2)	0.13	3.4 (1.7; 5.1)	**< 0.001**	0.11
Model 2	4.3 (2.4; 6.1)	**< 0.001**	7.6 (5.7; 9.5)	**< 0.001**	**0.02**	1.9 (− 0.0; 3.8)	0.06	3.7 (2.0; 5.5)	**< 0.001**	0.10
**Lactosylceramides**										
LacCer(d18:1)										
Model 1	0.3 (− 8.5; 9.2)	0.94	9.1 (0.5; 17.6)	**0.04**	0.16	− 7.9 (− 17.4; 2.1)	0.12	− 11.1 (− 18.7; − 3.6)	**0.004**	0.58
Model 2	1.6 (− 8.4; 11.5)	0.64	12.7 (3.6; 21.8)	**0.007**	0.19	− 6.5 (− 17.1; 4.1)	0.23	− 11.2 (− 19.3; − 3.1)	**0.007**	0.58
LacCer(d18:2)										
Model 1	1.4 (0.1; 2.6)	**0.03**	4.6 (3.2; 5.9)	**< 0.001**	**0.001**	− 0.1 (− 1.4; 1.1)	0.86	1.2 (0.0; 2.4)	**0.04**	0.12
Model 2	1.4 (0.0; 2.8)	**0.05**	4.8 (3.4; 6.3)	**< 0.001**	**0.001**	0.0 (− 1.3; 1.4)	0.94	1.2 (0.0; 2.5)	0.06	0.13
**Globotriaosylceramides**										
CTH(d18:1)										
Model 1	2.3 (− 0.6; 5.1)	0.12	5.2 (1.8; 8.6)	**0.003**	0.20	− 0.4 (− 3.6; 2.7)	0.79	1.2 (− 1.5; 3.9)	0.39	0.44
Model 2	3.6 (0.4; 6.8)	**0.03**	7.2 (3.7; 10.8)	**< 0.001**	0.24	1.0 (− 2.3; 4.4)	0.13	2.4 (− 0.5; 5.2)	0.10	0.33
CTH(d18:2)										
Model 1	1.0 (0.4; 1.6)	**0.002**	1.8 (1.0; 2.6)	**< 0.001**	0.14	0.2 (− 0.5; 0.9)	0.61	1.2 (0.4; 2.0)	**0.003**	0.07
Model 2	1.1 (0.4; 1.8)	**0.002**	2.1 (1.2; 2.9)	**< 0.001**	0.18	0.4 (− 0.4; 1.2)	0.29	1.3 (0.4; 2.2)	**0.003**	0.08

Model 1 shows the unadjusted increase in sphingolipid concentrations by age, while model 2 was adjusted for BMI and waist circumference

Sensitivity analyses with additional adjustment for use of cholesterol- or blood pressure-lowering medication, plasma amino acid (serine, alanine, glycine) concentrations, and energy or fat intake did not alter the results (data not shown). The sensitivity analyses excluding participants with CVD also did not change our interpretations (data not shown). Additional adjustment for menopause did, overall, not alter our interpretation (Supplementary Table 1). Menopause, however, mediated the associations between age and Cer(d18:0) and Cer(m18:0).

## Discussion

Our study shows that plasma levels of sphingolipids are generally lower among women than men in younger age categories, while they are higher among women than men in older age categories. Most sphingolipid concentrations increase by age in both men and women, but increases by age are larger in women than in men. Adiposity levels decreased the strength of observations, but did not impact the observed patterns of sex differences.

Studies on sex differences in sphingolipid concentrations showed conflicting results. A study by Sui et al. suggests that lactosylceramide concentrations are higher among women than men [19], whereas a study by Ishikawa et al. suggested generally similar levels of sphingolipids in both sexes [39], and a study by Weir et al. higher ceramide concentrations among men than women [29]. These studies, however, did not consider that the difference in concentrations by sex may differ by age, and the included study populations in the studies by Ishikawa et al. and Weir et al. were approximately 10 years younger than those in the study by Sui et al [19, 29, 39]. A study by Mielke et al. did consider age groups and showed that ceramide and dihydroceramides concentrations were higher in women than in men and increased more strongly in women than in men by age [30]. Although the study by Mielke et al. was limited to participants over 55 years of age [30], this finding is in line with our study. We added to these findings that the slope of the association is such that in younger age groups sphingolipid concentrations may be higher among men than women. Moreover, we are the first to report on sex differences in 1-deoxyceramides, glucosylceramides, and globotriaosylceramides, for which patterns of sex differences and age trends were similar to the dihydroceramides.

The higher levels of sphingolipids in (especially older) women when compared to men may partly be explained by a drop in oestrogen levels after menopause in women [40]. The drop in oestrogen levels may lead to a change in body fat distribution; however, adjustments for adiposity levels suggest that differences in body fat distribution are, apparently, not strongly related to sex differences in sphingolipid concentrations. Although it was in contrast with our hypothesis, sphingolipids are associated with the amount of visceral fat [41], and (especially younger) men are more likely to store visceral fat than women. Perhaps, measures of adiposity used in our study (BMI and waist circumference) do not properly reflect the difference in amounts of visceral fat between men and women [42]. However, post-hoc adjustments for menopause also did not support a major role for specific changes after menopause. Menopause only mediated the associations between age and the saturated (18:0) sphingolipid species in our analyses, while especially the mono-unsaturated species showed steeper increases in women than in men. Other mechanisms may also explain the observed sex difference in sphingolipid concentrations. One of the obvious differences between men and women are sex steroid differences. Already in 1985, studies showed that estradiol levels were associated with reduced concentrations of sphingolipids, but only in women [43], possibly by downregulation of key enzymes for de novo ceramide synthesis such as serine-palmitoyltransferase and ceramide synthase. Another possible candidate mechanism includes oxidative stress leading to inflammation and higher sphingolipid concentrations, which could for instance lead to CVD and T2D [12]. Generally, levels of oxidative stress were observed to be lower among women than men, but higher among post-menopausal women [44]. Finally, the higher sphingolipid concentrations among women than men may also be explained by differences in lipid lipoprotein metabolism, especially the higher levels of high density lipoproteins (HDL) particles in women [45], since lipoproteins transport insoluble lipids such as sphingolipids in the circulation.

Ceramides have been implicated in the development of age-related diseases including T2D and cardiovascular disease, for instance by inducing insulin resistance, formation of plaques, pro-inflammatory properties, and apoptosis [13–22]. We showed that ceramide concentrations increase with age, more in women than in men. The complex sphingolipids, associated with decreased T2D risk [22, 23], also increased with age, which may be explained by the fact that ceramides are precursors for these more complex sphingolipids. Nonetheless, age may not affect all sphingolipid concentrations similarly since sphingolipid metabolic pathways are highly complex and contain many different enzymes which may be differently affected by age [46]. This is also underscored by the observed increase of specific sphingolipid species concentration with age in women than in men, since the steeper increase was especially observed for the d18:2 sphingolipid species. The d18:1 sphingolipid species are formed by condensation of palmitoyl-CoA (C16:0) with serine by serine palmitoyl transferase (SPT) followed by DEGS-dependent desaturation of the sphinganine backbone at the dihydroceramide stage. The d18:2 sphingolipid species are formed similarly, but palmitoleic acid (C16:1) condenses with serine, later followed by DEGS desaturation. We speculate that the higher d18:2 levels in women could be caused by a higher dietary intake of palmitoleic acid or a higher stearoyl-CoA desaturase (SCD1) activity, which forms the n-9 double bond in activated saturated fatty acids (C16:0). Whether this increase in d18:2 species is linked to disease and what mechanism causes this remains to be established.

Our study is not exempt from limitations. First, our study is a secondary analysis of existing data. In the sampling procedure participants with T2D were excluded from the study. This may have affected our results since sphingolipids are associated with T2D [20], underestimating the mean concentrations across groups. This may especially have affected the results for men and the South-Asian Surinamese participants, since T2D is more prevalent among men and those of South-Asian descent [47, 48]. Further, our study included only participants of two ethnic groups, and findings need to be replicated among participants from other ethnic backgrounds, especially since the strength of associations differed by ethnicity. Nevertheless, patterns of differences were consistent across both ethnic groups although the sex difference in age-related increase of sphingolipids was more apparent among the Dutch. In addition, sensitivity analyses that also excluded participants with baseline CVD, also more prevalent among those of South-Asian descent than in the majority Dutch, did not affect our results. Next, the cross-sectional design of our study is a limitation. We did not follow participants over time, but cross-sectionally grouped our study population by age. The results may thus reflect a cohort effect. Characteristics of older participants may differ from younger participants, which is especially important if characteristics of women have changed differently over time than those of men. This seems unlikely, since a linear association between age and sphingolipids was observed; this is only likely if characteristics have changed gradually over time. Nevertheless, longitudinal studies are needed to confirm our findings.

Plasma sphingolipid levels increase with age in both men and women. While sphingolipids are lower in young women than men, the sphingolipids increase more rapidly with age in women than men, leading to higher sphingolipid levels in women than men at higher age. A better understanding of sex differences in age-related trajectories of sphingolipids is important since sphingolipids have repeatedly been associated with age-related diseases. Future studies may investigate whether the observed changes in sphingolipid concentrations by age are reflective of other processes and may serve as biomarkers for disease risk or are a target in itself to reduce CVD risk. This understanding may help in developing targeted interventions and to identify biomarkers for disease risk.

## Supplementary Information

**Additional file 1: Supplementary Table 1.** Association of sphingolipids with age, additionally adjusted for menopause.

**Additional file 2:** Sphingolipid concentrations by age, stratified by sex and ethnicity.

## Data Availability

The HELIUS data are owned by the Academic Medical Center (AMC) in Amsterdam, The Netherlands. Any researcher can request the data by submitting a proposal to the HELIUS Executive Board as outlined at http://www.heliusstudy.nl/en/researchers/collaboration. Requests for further information and proposals can be submitted to the Scientific Coordinator and Data Manager of HELIUS, at info@heliusstudie.nl. The HELIUS Executive Board will check proposals for compatibility with the general objective, ethical approvals, and informed consent forms of the HELIUS study, and potential overlap with ongoing work affiliated with HELIUS. There are no other restrictions to obtaining the data, and all data requests will be processed in the same manner.
