# Supplementary material for: Sex differences in the association of sphingolipids with age in Dutch and South-Asian Surinamese living in Amsterdam, the Netherlands
Source: Biol Sex Differ. 2021 Jan 13;12:13. doi: 10.1186/s13293-020-00353-0 (PMC7805203; doi:10.1186/s13293-020-00353-0)
Supplement: Supplementary file 1 — Additional file 1: Supplementary Table 1. Association of sphingolipids with age, additionally adjusted for menopause. [file 13293_2020_353_MOESM1_ESM.docx]

*Supplementary Table 1: Association of sphingolipids with age, additionally adjusted for menopause*

| **Sphingolipid (nMol/L)** | **Dutch men (n=174)** |  | **Dutch women (N=176)** |  | **SA Sur men (N=154)** |  | **SA Sur women (N=196)** |  |
| --- | --- | --- | --- | --- | --- | --- | --- | --- |
|  | **B (95% CI)** | **p-value** | **B (95% CI)** | **P-value** | **B (95% CI)** | **p-value** | **B (95% CI)** | **P-value** |
| **Ceramides** |  |  |  |  |  |  |  |  |
| Cer(d18:1) Model 2 | 31 (2; 59) | **0.03** | 76 (55; 98) | **<0.001** | 33 (4; 61) | **0.03** | 31 (10; 53) | **0.005** |
| Model 2 + menopause |  |  | 49 (15.4; 83) | **0.005** |  |  | 36 (10; 62) | **0.006** |
| Cer(d18:2) Model 2 | 3.2 (-2.1; 8.5) | 0.23 | 15.9 (11.6; 20.3) | **<0.001** | 8.3 (2.7; 14.0) | **0.004** | 9.9 (5.4 14.5) | **<0.001** |
| Model 2 + menopause |  |  | 11.3 (4.4; 18.2) | **0.001** |  |  | 9.6 (4.2; 15.1) | **0.001** |
| Cer(d18:0) Model 2 | 2.2 (-4.7; 9.1) | 0.53 | 5.7 (-1.0; 12.3) | 0.09 | 6.6 (-2.4; 15.7) | 0.15 | 3.2 (-4.2; 10.5) | 0.40 |
| Model 2 + menopause |  |  | 0.5 (-10.0; 11.1) | 0.92 |  |  | 2.81 (-5.9; 11.6) | 0.53 |
| Cer(d16:1) Model 2 | 1.1 (-1.3; 3.6) | 0.37 | 5.3 (3.4; 7.3) | **<0.001** | 4.7 (2.1; 7.3) | **<0.001** | 3.8 (1.9; 5.6) | **<0.001** |
| Model 2 + menopause |  |  | 2.9 (-0.2; 6.1) | **0.06** |  |  | 3.0 (0.8; 5.2) | **0.007** |
| Cer(d17:1) Model 2 | 1.7 (-0.0; 3.4) | 0.06 | 4.1 (2.7; 5.4) | **<0.001** | 2.4 (0.8; 4.0) | **0.003** | 2.3 (1.1; 3.4) | **<0.001** |
| Model 2 + menopause |  |  | 2.4 (0.3; 4.5) | **0.03** |  |  | 1.9 (0.5; 3.3) | **0.008** |
| Cer(d20:1) Model 2 | 0.79 (-0.04; 1.61) | 0.06 | 0.88 (-0.34; 2.11) | 0.16 | 0.09 (-0.91; 1.09) | 0.86 | 0.71 (-0.10; 1.53) | 0.09 |
| Model 2 + menopause |  |  | 0.94 (-1.02; 2.90) | 0.35 |  |  | 0.42 (-0.54; 1.38) | 0.39 |
| **1-deoxysphinganine** |  |  |  |  |  |  |  |  |
| Cer(m18:0) Model 2 | 2.2 (-4.7; 9.1) | 0.53 | 5.7 (-1.0; 12.3) | 0.09 | 6.6 (-2.4; 15.7) | 0.15 | 3.2 (-4.2; 10.5) | 0.40 |
| Model 2 + menopause |  |  | 0.1 (-0.1; 0.3) | 0.38 |  |  | 0.1 (-0.1; 0.2) | 0.34 |
| **Glucosylceramides** |  |  |  |  |  |  |  |  |
| GlcCer(d18:1) Model 2 | 21 (8; 34) | **0.002** | 40 (27; 52) | **<0.001** | 6 (-9; 20) | 0.44 | 5 (-6; 16) | 0.38 |
| Model 2 + menopause |  |  | 41 (21; 61) | **<0.001** |  |  | 7 (-6; 20) | 0.32 |
| GlcCer(d18:2) Model 2 | 4.3 (2.4; 6.1) | **<0.001** | 7.6 (5.7; 9.5) | **<0.001** | 1.9 (-0.0; 3.8) | 0.06 | 3.7 (2.0; 5.5) | **<0.001** |
| Model 2 + menopause |  |  | 6.3 (3.4; 9.3) | **<0.001** |  |  | 3.7 (1.6; 5.9) | **0.001** |
| **Lactosylceramides** |  |  |  |  |  |  |  |  |
| LacCer(d18:1) Model 2 | 1.6 (-8.4; 11.5) | 0.64 | 12.7 (3.6; 21.8) | **0.007** | -6.5 (-17.1; 4.1) | 0.23 | -11.2 (-19.3; -3.1) | **0.007** |
| Model 2 + menopause |  |  | 16.4 (1.8; 30.9) | **0.03** |  |  | -11.1 (-20.7; -1.4) | **0.03** |
| LacCer(d18:2) Model 2 | 1.4 (0.0; 2.8) | **0.05** | 4.8 (3.4; 6.3) | **<0.001** | 0.0 (-1.3; 1.4) | 0.94 | 1.2 (0.0; 2.5) | 0.06 |
| Model 2 + menopause |  |  | 4.1 (1.8; 6.4) | **0.001** |  |  | 1.2 (-0.3; 2.7) | 0.12 |
| **Globotriaosylceramides** |  |  |  |  |  |  |  |  |
| CTH(d18:1) Model 2 | 3.6 (0.4; 6.8) | **0.03** | 7.2 (3.7; 10.8) | **<0.001** | 1.0 (-2.3; 4.4) | 0.13 | 2.4 (-0.5; 5.2) | 0.10 |
| Model 2 + menopause |  |  | 5.3 (-0.3; 11.0) | **0.07** |  |  | 3.6 (0.2; 7.0) | 0.04 |
| CTH(d18:2) Model 2 | 1.1 (0.4; 1.8) | **0.002** | 2.1 (1.2; 2.9) | **<0.001** | 0.4 (-0.4; 1.2) | 0.29 | 1.3 (0.4; 2.2) | **0.003** |
| Model 2 + menopause |  |  | 1.0 (-0.4; 2.4) | **0.15** |  |  | 1.4 (0.3; 2.4) | **0.01** |

*Model 2 was adjusted for BMI and waist circumference, and the analyses for women were additionally adjusted for menopause.*
