# Supplementary figures and images for: Sex differences in the association of sphingolipids with age in Dutch and South-Asian Surinamese living in Amsterdam, the Netherlands
Source: Biol Sex Differ. 2021 Jan 13;12:13. doi: 10.1186/s13293-020-00353-0 (PMC7805203; doi:10.1186/s13293-020-00353-0)

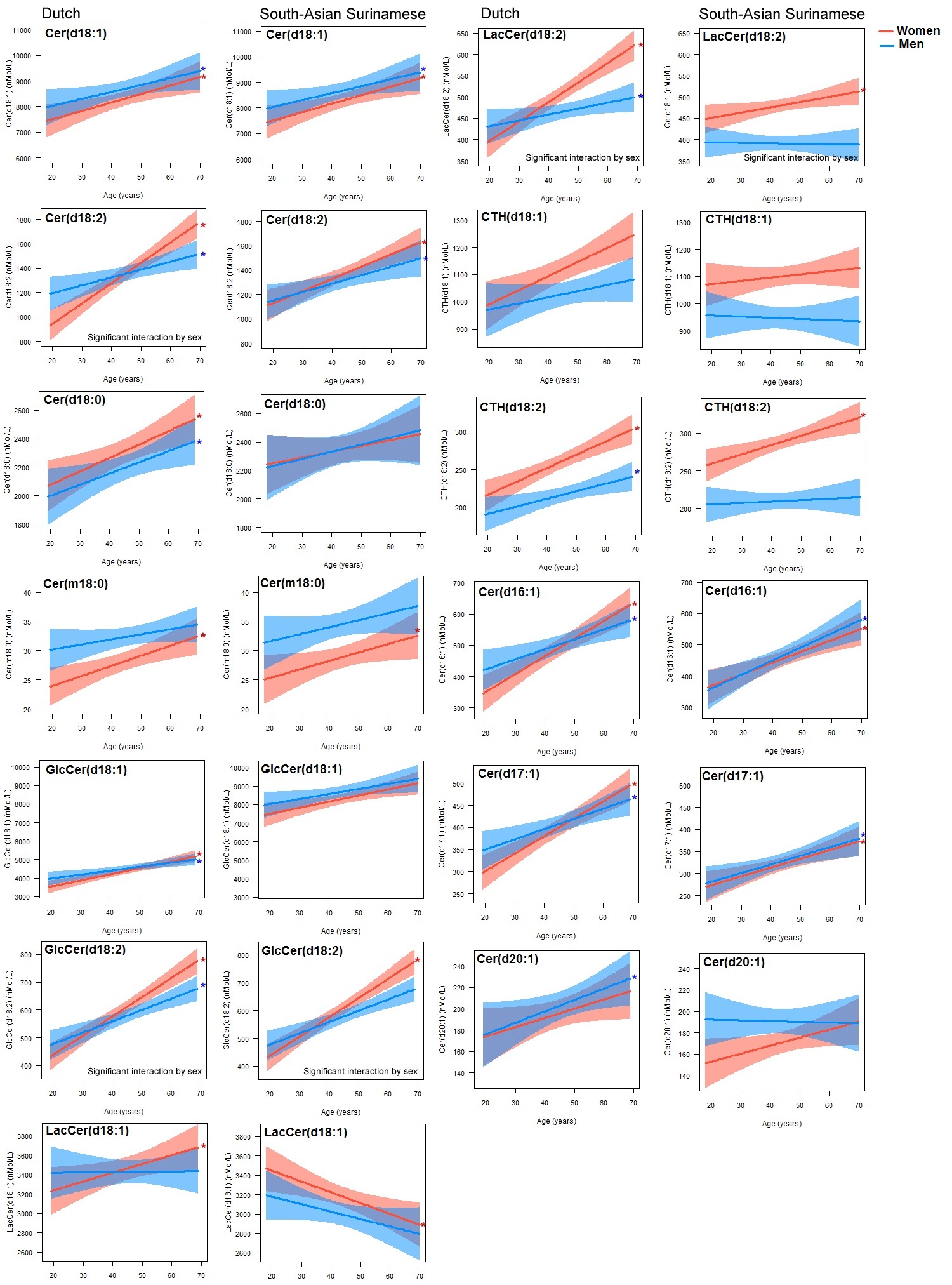

Supplement: Supplementary file 2 — Additional file 2: Sphingolipid concentrations by age, stratified by sex and ethnicity. [file 13293_2020_353_MOESM2_ESM.jpg]
